# Genetic Diversity of *Mycobacterium tuberculosis* Strains Isolated from HIV-Infected Patients in Mexico

**DOI:** 10.3390/pathogens13050428

**Published:** 2024-05-19

**Authors:** Daniel Valencia-Trujillo, Amanda Marineth Avila-Trejo, Rocío Liliana García-Reyes, Luis Narváez-Díaz, Mariela Segura del Pilar, Mario Alberto Mújica-Sánchez, Eduardo Becerril-Vargas, Moises León-Juárez, Mónica Maribel Mata-Miranda, Sandra Rivera-Gutiérrez, Jorge Francisco Cerna-Cortés

**Affiliations:** 1 Departamento de Microbiología, Escuela Nacional de Ciencias Biológicas, Instituto Politécnico Nacional, Ciudad de México 11340, Mexico; vatd921205@hotmail.com (D.V.-T.); rlgarciar@ipn.mx (R.L.G.-R.); srivera@ipn.mx (S.R.-G.); 2 Servicio de Microbiología Clínica, Instituto Nacional de Enfermedades Respiratorias, Ciudad de México 14080, Mexico; nluisnd0304@hotmail.com (L.N.-D.); mariela88.sp@gmail.com (M.S.d.P.); mario.mugica1@gmail.com (M.A.M.-S.); edobec.var@gmail.com (E.B.-V.); 3 Escuela Militar de Medicina, Centro Militar de Ciencias de la Salud, Secretaría de la Defensa Nacional, Ciudad de México 11200, Mexico; ammarat.gdds@gmail.com (A.M.A.-T.); mmcmaribel@gmail.com (M.M.M.-M.); 4 Departamento de Inmunobioquímica, Instituto Nacional de Perinatología Isidro Espinosa de los Reyes, Ciudad de México 11000, Mexico; moisesleoninper@gmail.com

**Keywords:** *Mycobacterium tuberculosis*, *tuberculosis*, genetic diversity, HIV-infected patients

## Abstract

There has been very limited investigation regarding the genetic diversity of *Mycobacterium tuberculosis* (MTb) strains isolated from human immunodeficiency virus (HIV)-infected patients in Mexico. In this study, we isolated 93 MTb strains from pulmonary and extrapulmonary samples of HIV-infected patients treated in a public hospital in Mexico City to evaluate the genetic diversity using spoligotyping and mycobacterial interspersed repetitive unit-variable-number tandem-repeat (MIRU-VNTR) typing (based on 24 loci). The cohort comprised 80 male and 13 female individuals. There was a positive correlation between a high HIV viral load (>100,000 copies) and extrapulmonary tuberculosis (TB) (*r* = 0.306, *p* = 0.008). Lineage 4 was the most frequent lineage (79 strains). In this lineage, we found the H clade (*n* = 24), including the Haarlem, H3, and H1 families; the T clade (*n* = 22), including T1 and T2; the X clade (*n* = 15), including X1 and X3; the LAM clade (*n* = 14), including LAM1, LAM2, LAM3, LAM6, and LAM9; the S clade (*n* = 2); Uganda (*n* = 1); and Ghana (*n* = 1). We also found 12 strains in the EAI clade belonging to lineage 1, including the EAI2-Manila and EAI5 families. Interestingly, we identified one strain belonging to the Beijing family, which is part of lineage 2. One strain could not be identified. This study reports high genetic diversity among MTb strains, highlighting the need for a molecular epidemiological surveillance system that can help to monitor the spread of these strains, leading to more appropriate measures for TB control in HIV-infected patients.

## 1. Introduction

Tuberculosis (TB) is a preventable and usually curable disease. However, by 2022, TB had become the world’s second leading cause of death from a single infectious agent, following the outbreak of coronavirus disease 2019 (COVID-19). More than 10 million people continue to fall ill with TB every year, and about a quarter of the global population is estimated to have been infected with the bacterium that causes TB [1]. TB is caused by the bacillus *Mycobacterium tuberculosis* (MTb), which spreads when individuals afflicted with TB expel bacteria into the air through actions such as coughing. Such aerosols can remain airborne and infectious for several hours, be carried in the air, and accumulate in poorly ventilated environments [2]. The disease typically affects the lungs (pulmonary TB), but it can also affect other sites. Active TB develops in approximately 5% of MTb-infected individuals within 2 years. In 90–95% of patients, dormant viable bacteria survive for years, progressing to active disease at a rate of 10% per lifetime in the human immunodeficiency virus (HIV)-uninfected population and at 10% per annum in HIV-infected patients [3]. Of the total number of people who develop TB each year, about 90% are adults, with more cases among men than women [1]. Without treatment, the death rate from TB is high (about 50%). In 2022, the World Health Organization (WHO) reported a total of 1.3 million deaths worldwide due to TB [1].

HIV-associated TB remains a major public health concern. TB is the leading HIV-associated opportunistic infection and the main cause of death globally, particularly in resource-limited settings [3]. Globally, HIV-associated TB was estimated to be responsible for about 6.3% of all new cases and 12.8% of all TB-related deaths [1]. Encouragingly, there has been a gradual decline in the prevalence of HIV among new TB cases in recent years [1]. Nevertheless, in Mexico, the prevalence of HIV among new TB cases has increased in recent years, going from 3500 in 2019 to 4500 in 2022 [4]. Additionally, the number of deaths due to TB among the HIV-positive population in Mexico has tripled in the same period [5]. Therefore, it is important to evaluate the genetic diversity of MTb strains to identify genotypes that affect HIV-infected patients. Indeed, determining the genetic diversity of MTb strains has emerged as a crucial public health tool, aiding researchers and TB control programs in comprehending the emergence and spread of specific strains, and assessing the overall impact of genetic diversity on the progression of TB infection and disease [6]. Furthermore, strains of different genetic lineages of MTb demonstrate variability in some biological properties such as the in vitro growth rate, virulence in animal models, and the capacity to acquire drug resistance [7].

The use of tools that allow for the evaluation of the genetic diversity of MTb strains, such as mycobacterial interspersed repetitive unit-variable-number tandem-repeat (MIRU-VNTR) typing based on 24 loci and spacer oligonucleotide typing (spoligotyping), are important for understanding the dynamics and complexity of the population structure of MTb within a population [8]. Spoligotyping detects variations in the direct-repeat (DR) locus, which consists of a repeated 36-base pair (bp) sequence interspersed with nonrepetitive 31–41 bp DNA segments called spacer sequences. The DR region is amplified by polymerase chain reaction (PCR), and the amplicon is hybridized to probes that detect the specific sequences of the spacers. A specific pattern of recognition of the spacers is called a spoligotype [9]. Spoligotyping has gained international approval as a robust, fast, and reproducible typing method suitable for epidemiological research [10]. MIRU-VNTR is a PCR-based typing method that determines the size and number of repeated units in each locus by amplifying the mycobacterial interspersed repetitive units [11]. Currently, MIRU-VNTR typing based on 24 loci is used the most; the increased number of analyzed loci has improved the discriminatory power compared with the original 12-locus set [12].

In Mexico, there has been very little investigation regarding genetic diversity among MTb strains isolated from HIV-infected patients [13,14,15]. To contribute to this knowledge, we evaluated the genetic diversity of MTb strains isolated from a group of HIV-infected patients in Mexico using spoligotyping and MIRU-VNTR typing based on 24 loci, with the overall aim of monitoring genetic diversity of these microorganisms and generate information for developed of public health interventions for HIV-infected patients.

## 2. Materials and Methods

### 2.1. Sample Collection and Study Population

**Figure 1:**
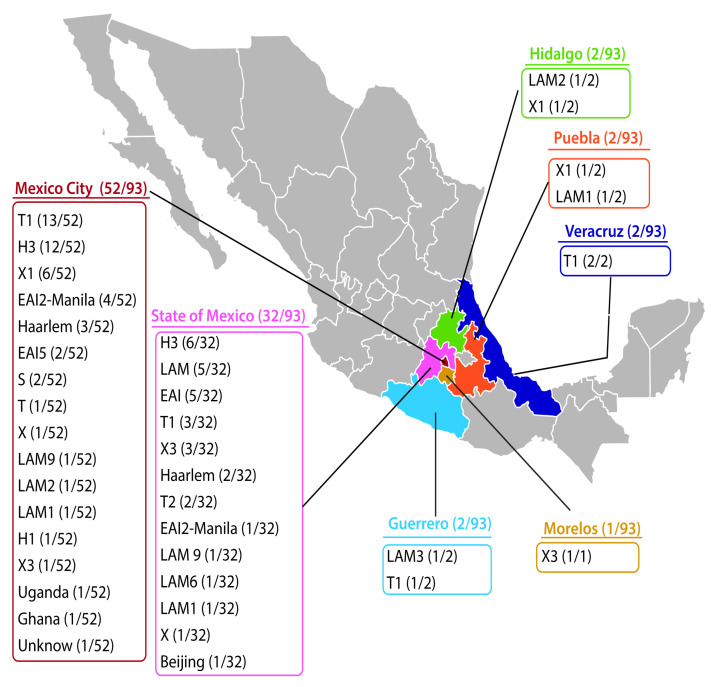
Geographic localization of each mycobacterial family identified in this study (central Mexico).

Ninety-three MTb strains isolated from the same number of HIV-infected patients were studied. The patients come from seven different states of Mexico (Figure 1) and were treated at the National Institute of Respiratory Diseases “Ismael Cosio Villegas” (Mexico City) between January 2014 and December 2019. The population included in this study were patients with confirmed HIV status, without any other comorbidities and with complete clinical records.

### 2.2. DNA Isolation and MTb Identification

Biological samples were taken as part of routine diagnostics. Samples were decontaminated using the modified Petroff method [16]. An aliquot was inoculated into Middlebrook 7H9 broth in the BACTEC™ MGIT™ automated mycobacterial detection system (BD, Sparks, MD, USA). Blood and bone marrow samples were not decontaminated and were directly inoculated into the BD BACTEC Myco/F Lytic culture vials (BD, Sparks, MD, USA), which were incubated in the BD BACTEC™ FX blood culture system (BD, Sparks, MD, USA). DNA was extracted and evaluated with the GenoType MTBC (HAIN Lifescience, Nehren, Germany), following the manufacturer’s instructions, to ensure reliable identification of MTb.

### 2.3. Spoligotyping

Spoligotyping was carried out following standard techniques [17,18]. The DR region was amplified using the oligonucleotides DRa (5′-GGTTTTGGGTCTGACGAC-3′, biotinylated) and DRb (5′-CCGAGAGGGGACGGAAAC′-3′). Labeled amplification products were used as a probe for hybridization with 43 synthetic spacer oligonucleotides covalently bound to a membrane (Isogen Biosciences B.M., Maarssen, The Netherlands). Each oligonucleotide corresponds to a known spacer sequence. The PCR product bound after hybridization was detected by streptavidin–horseradish peroxidase-enhanced chemiluminescence. The membrane was exposed to a chemiluminescence system, followed by exposure to X-ray film (Amersham, Little Chalfont, UK) according to the manufacturer’s instructions. The spoligotypes are reported using an octal code [19]. They were analyzed using the Bionumerics software version 5.5 (Applied Maths, Kortrijk, Belgium). MTb H37Rv was used as a control. The lineage and family were assigned according to the SITVIT2 database (http://pasteur-guadeloupe.fr:8081/SITVIT2/, version β 2.0, Guadeloupe, France, accessed on 10 July 2023) [20]. The strains that did not match any preexisting patterns in the SITVIT2 database were individually identified using the similarity search module of the MIRU-VNTRplus database (http://www.miru-vntrplus.org/MIRU/index.faces, software version 1.0.22, Borstel, Germany, accessed on 12 July 2023). The distance value was between the strict value (0.05) and the relaxed value (0.1), as other authors have reported [21], except for the values 0.1395, 0.14, and 0.16 used at strains INER-87, INER-93 and INER-20, respectively, considering that using a value between the relaxed value to 0.3 the specificity of identification range from 95.7 to 96.2%. A posteriori visualization of the corresponding spoligotype on the tree was used to assign the family. A posteriori visualization of the corresponding spoligotype on a tree constructed using reference strains from the database was used to confirm the family [22]. The spoligotype patterns were used to construct a dendrogram with the neighbor-joining algorithm using the online MIRU-VNTRplus application after pairwise comparison of strains based on the Jaccard index.

### 2.4. MIRU-VNTR Typing

MIRU-VNTR typing based on 24 loci was conducted following the recommendations of Supply et al. [23]. Each locus was amplified individually by PCR followed by the determination of the size of the amplicon by electrophoresis using a 2% agarose. Fragment size was estimated by comparison with 100 bp DNA molecular weight ladders (Invitrogen^TM^, Carlsbad, CA, USA). Since the length of the repeat units is known, the calculated size reflects the numbers of the amplified MIRU copies. The final result is a multidigit numerical code number corresponding to the repeat number at each analyzed locus. The MIRU-VNTRplus application was used to construct a minimal spanning tree based on the MIRU-VNTR data from 24 loci. The maximum locus difference within a clonal complex was double the locus variants (DLV2), and the circle size indicates the frequency. The discriminatory power of MIRU-VNTR typing was measured for each locus using a calculator (insilico.ehu.eus), based on the Hunter–Gaston index (HGDI) [24].

### 2.5. Statistical Analysis

GraphPad Prism 8.0.2 (GraphPad Software, San Diego, CA, USA) was used for statistical analysis. All data were analyzed with the Shapiro–Wilk and Kolmogorov–Smirnov tests to assess the distribution. The Spearman rho test was used to identify correlations between clinical and sociodemographic characteristics and spoligotype and MIRU-VNTR patterns. A *p* value < 0.05 was considered to be statistically significant.

## 3. Results

### 3.1. Sociodemographic and Clinical Characteristics of the Patients

We analyzed 93 MTb strains isolated from the same number of HIV-infected patients from seven states of Mexico (Figure 1). The cohort comprised 80 male and 13 female individuals. Sixty-eight (73%) patients had extrapulmonary TB, and the remaining had pulmonary TB. Of these 93 patients, 82.7% were new TB cases. The age ranged from 7 and 65 years, with an average of 36 years. The CD4 T lymphocyte count was quantified in 69 patients: for 51 patients, it was <350 cells/mm^3^, and for the remaining 18 patients it was >350 cells/mm^3^. Ninety-four percent of patients with extrapulmonary TB had a CD4 T lymphocyte count <350 cells/mm^3^, while seventy-eight percent of patients with pulmonary TB had a CD4 T lymphocyte count <350 cells/mm^3^. In 73 patients, the HIV viral load was determined: eight patients had an undetectable viral load, 32 patients had <100,000 copies, and the remaining 33 patients had a high viral load (>100,000 copies). There was a positive correlation between a high HIV viral load (>100,000 copies) and extrapulmonary TB (*r* = 0.306, *p* = 0.008). Mycobacteria were isolated from both pulmonary and extrapulmonary samples: bronchoalveolar lavage fluid (*n* = 17), sputum (*n* = 15), lung biopsies (*n* = 6), blood (*n* = 15), bone marrow (*n* = 11), urine (*n* = 8), cervical lymph node (*n* = 8), pleural effusion (*n* = 6), subcutaneous abscesses (*n* = 4), and cerebrospinal fluid (*n* = 3).

### 3.2. Spoligotyping

**Figure 2:**
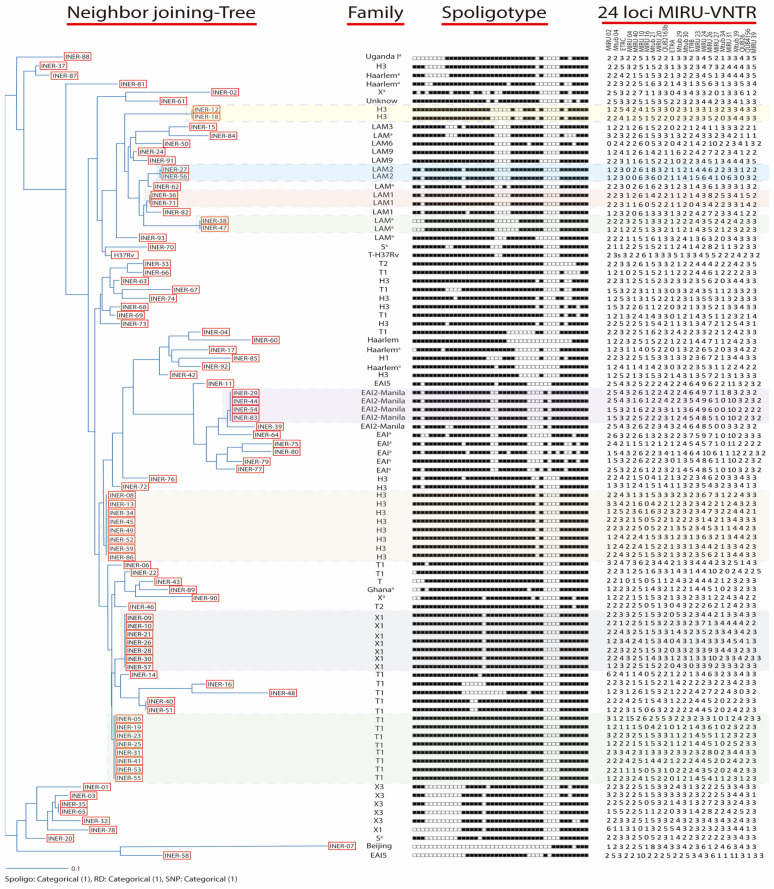
Dendrogram of the 93 MTb strains isolated from HIV-infected patients, along with the family, spoligotype patterns, and MIRU-VNTR patterns (based on 24 loci). The phylogenetic tree was generated from the spoligotype patterns. ^a^ Family identified using the MIRU-VNTR plus database.

From the 93 MTb strains, there were 66 different spoligotype patterns. Based on the dendrogram (Figure 2), 35 strains were grouped into 8 clusters, and 58 strains had single patterns. Seventy-one MTb strains matched 40 spoligotype international types (SITs) in the SITVIT2 database (Table 1). One pattern was not classified with an SIT, but it was identified as T1. Twenty-one patterns from 21 strains (one for each strain) did not match any preexisting patterns in the SITVIT2 database. We identified 20 of these strains with single patterns using the MIRU-VNTRplus database; we could not identify the INER-61 strain (Table 2). The most frequent lineage (79 strains) was lineage 4 (Euro-American). In this lineage, we found the H clade (*n* = 24), including the Haarlem, H3, and H1 families; the T clade (*n* = 22), including the T1 and T2 families; the X clade (*n* = 15), including the X1 and X3 families; the LAM clade (*n* = 14), including the LAM1, LAM2, LAM3, LAM6, and LAM9 families; the S clade (*n* = 2); Uganda (*n* = 1); and Ghana (*n* = 1). We also found 12 strains belonging to lineage 1 (Indo-Oceanic) in the EAI clade (*n* = 12), including EAI2-Manila and EAI5. Interestingly, we identified the Beijing family (*n* = 1) belonging to lineage 2. The geographic distribution of each family is presented in Figure 1. There was no correlation between the spoligotyping patterns and the clinical and sociodemographic characteristics.

### 3.3. MIRU-VNTR Typing

**Figure 3:**
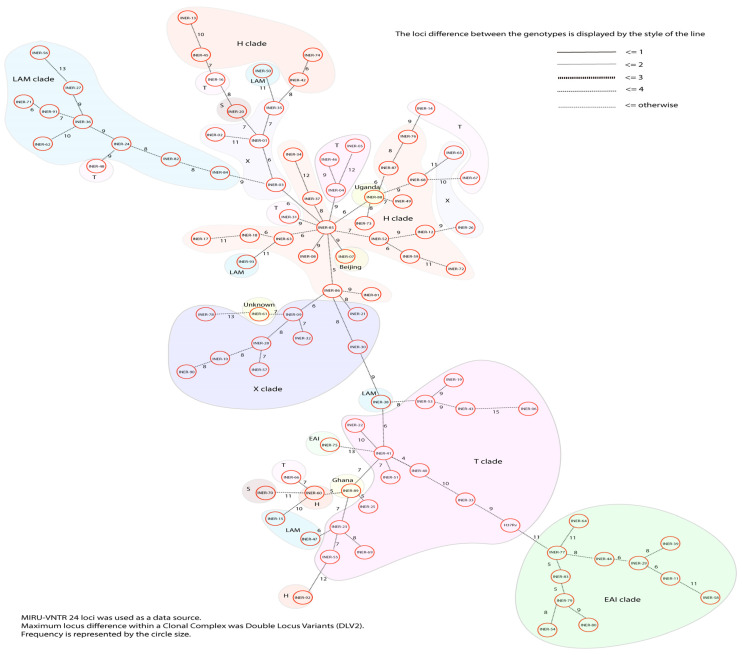
The minimal spanning tree of the 93 MTb strains based on the MIRU-VNTR patterns (24 loci). The clades have been colored for illustrative purposes.

From the 93 MTb strains, we obtained a total of 93 different MIRU-VNTR patterns, one for each strain (Figure 2). We also generated a minimal spanning tree (Figure 3). Both results indicated no epidemiological relationships among the strains: There was no cluster formation in the dendrogram or clonal complex formation in the minimal spanning tree when we analyzed the MIRU-VNTR patterns of the strains. The MIRU-VNTR patterns show the low transmissibility of MTb strains and their greater discriminatory power compared with spoligotyping. The closest strains were INER-41 and INER-40, showing four allelic differences; the other strains had at least five differences. Based on the HGDI, the combination of the 24 loci had a total resolution index of 1. We evaluated the allelic diversity of each MIRU-VNTR locus based on the HGDI (Table 3). The HGDI of 17 loci exceeded 0.6, classifying them as highly discriminating loci. The other seven loci showed moderate discrimination (0.3 < HGDI < 0.6). We observed greater polymorphism in MIRU 24 and 26. There was no correlation between the MIRU-VNTR patterns and the clinical and sociodemographic characteristics.

## 4. Discussion

In recent years, MTb genotyping has been a useful tool for epidemiological research regarding the dynamics of transmission in certain regions. Several studies have reported an association between lineages and their propensity to be transmitted and cause disease [25]. In this study, we determined the genetic diversity of 93 MTb strains isolated from pulmonary and extrapulmonary samples of HIV-infected patients using two PCR-based methods to monitor the spread of these microorganisms in the HIV-infected population. We analyzed a total of 93 strains from same number of HIV-positive patients. With the exception of 8 patients, the remaining 65 presented an HIV viral load because they had recently been diagnosed with HIV; therefore, the patients had not received antiretroviral treatment. We found a positive correlation between a high viral load and extrapulmonary TB. It has been reported that HIV-positive patients with ongoing HIV replication, as determined by plasma HIV viral loads, have an increased risk for TB, independent of the CD4 cell count [26]. Moreover, in HIV-positive patients, MTb exacerbates the viral load, and HIV coinfection can alter the pathogenesis of MTb and lead to negative sputum smear results, atypical radiographic manifestations, and extrapulmonary manifestations, which pose difficulty in diagnosing TB [27].

The combined use of spoligotyping and MIRU-VNTR typing based on 24 loci allowed us to identify the genetic diversity among the 93 identified MTb strains. The predominant lineage in this study was lineage 4 (*n* = 79), followed by lineage 1 (*n* = 12) and lineage 2 (*n* = 1). Our results are similar to those obtained by Ordaz-Vázquez et al. [28] in southern Mexico: They found a high frequency of lineage 4 followed by lineage 1 and lineage 2. Lineage 4 has been described as the most frequent in other studies from Mexico [8,9,29,30,31]. Lineage 1 was the second most abundant (*n* = 12), including the families EAI2-Manila and EAI5. These families have been frequently reported in countries between the Tropic of Cancer and the Equator and in Southeast Asia [32]. Moreover, lineage 1 has been reported in Latin America [33] and Mexico [34]. In our study, families belonging to lineage 1 were isolated from patients living in Mexico City and the State of Mexico, two places that have a lot of international contact and displacement of people. However, the relationship between the lineage 1 families and migratory or tourism activities should be analyzed in detail using more resolutive tools [31]. We also detected the Beijing family, belonging to lineage 2, which has been associated with high drug resistance, an increase in disease severity, less protection by the bacillus Calmette–Guérin (BCG) vaccine, and greater transmissibility [35]. This family has previously been reported in Mexico [29]; therefore, the prevalence of the Beijing strains in our country needs to be monitored to determine whether they are expanding.

The most frequent clade we found was H, followed by T, X, LAM, and EAI. Several studies have shown the distribution of clades and families across Mexico, and all these reports are consistent with our findings [8,29,30]. Flores-Treviño et al. [29] reported that the LAM clade was the most frequent among MTb strains isolated from pulmonary samples of patients from a western region of Mexico. Similarly, Flores-Lopez et al. [36] found that the LAM clade was the most frequent in a population from Baja California, Mexico. Of note, that study examined a border city where migratory phenomena are much more frequent and have an important role in the TB epidemiology of the region. The LAM clade has been reported as the most frequent in South America, especially in Venezuela, Colombia, Brazil, and Paraguay [37], coinciding with the migratory movements that have occurred in recent years.

In this study, 35 strains were grouped into 8 clusters, seven of which belonged to lineage 4. This lineage recently has been associated with cavitations and treatment failure [38]. The cluster remaining belonged to lineage 1, which can cause osteomyelitis among patients with extrapulmonary TB [39].

There are very few studies of the genetic diversity of MTb strains in HIV-infected patients in Mexico, and the ones that are available are not current. In 2010, Lopez-Alvarez et al. [15] evaluated 67 MTb strains from pulmonary and extrapulmonary samples of HIV-infected patients. They found 21 spoligotype patterns, and the T and LAM clades were the most frequent. Another study evaluated 97 MTb strains isolated from patients with different immunodeficiencies [14], including HIV, diabetes, chronic renal failure, and malnutrition, and also reported the LAM, H, and T clades as the most frequent.

The T1 and H3 families were the most frequent in our study, which is consistent with the report by Zenteno-Cuevas et al. [31] among drug-resistant MTb strains from Southeast Mexico. The X1 family was the third most frequent in our study; this family has also been reported in a study from Monterrey, Mexico [9]. We also found one strain that could not be classified and 21 spoligotype patterns that had not previously been reported in the SITVIT2 database. Likewise, Lopez-Avalos et al. [30] identified new spoligotype patterns among MTb strains from Jalisco, Mexico, suggesting that those strains are endemic to the region. More studies of genetic diversity are needed to identify endemic strains in Mexico.

The use of MIRU-VNTR typing based on 24 loci demonstrated a high discriminatory power and null transmissibility given that no clusters were formed. The high discriminatory power of MIRU-VNTR typing has been reported previously [28]. Our results showed greater polymorphism for ETRC, Mtub 04, Mtub 29, Mtub 24, Mtub 26, Mtub 34, and QUB26, findings that differ from the study by Cortés-Torres et al. [14], who reported that MIRU 10, MIRU 16, MIRU 23, and MIRU 27 presented greater polymorphism in MTb strains isolated from immunocompromised patients. These findings indicate that there is a high polymorphism in the MIRU in the immunosuppressed population. These characteristics could be useful for the identification and classification of MTb strains that most frequently affect immunocompromised patients. There was no correlation between the spoligotyping and MIRU-VNTR patterns with the clinical and sociodemographic characteristics, consistent with a previous report by Martinez-Guarneros et al. [40].

Our study has a few limitations. The incomplete clinical records we excluded from our analysis might introduce selection bias and decrease the number of people in the study population. It is important to consider that the entire population in this study was HIV positive. For future studies, a control group of HIV-negative patients with TB is needed.

In conclusion, we found high genetic diversity in MTb strains isolated from HIV-infected patients. We identified MTb strains that share a spoligotype pattern but do not have an epidemiological relationship, suggesting that transmissibility of these MTb strains is low in the studied population. We found some unmatched spoligotype patterns; a high frequency of the T, H, and LAM clades; and high polymorphism in some MIRU. These results will help to determine how these MTb families could be influenced by geographic and immunological factors and also improve the TB control program, allowing not only to focus on pulmonary TB, but also on extrapulmonary TB, permitting adequate management and implementation of public health interventions in this vulnerable population. This study adds to the knowledge of the genetic diversity in central Mexico; however, more studies are needed at the national level to evaluate the dynamics of TB transmission in this important vulnerable population. In Mexico, lineage determination is only performed for research purposes; therefore, we suggest that lineage determination should be performed as part of TB diagnosis and then create a molecular epidemiological surveillance system that allows monitoring the spread of these microorganisms, leading to more appropriate measures for TB control in HIV-infected patients.

## Figures and Tables

**Table 1:** The spoligotype international types (SITs), family, and number of strains found in this study using the SITVIT2 database.

SIT	Family	Number of Strains	SIT	Family	Number of Strains
7	T1	1	520	T1	1
17	LAM2	2	732	T1	2
19	EAI2-Manila	4	747	H3	1
20	LAM1	2	751	T	1
42	LAM9	1	1095	X1	1
50	H3	9	1170	EAI2-Manila	1
52	T2	1	1223	T1	1
53	T1	9	1274	Haarlem	1
70	X3	2	1328	H1	1
92	X3	2	1347	T1	1
95	LAM6	1	1611	H3	1
119	X1	7	1908	H3	1
161	LAM9	1	2111	H3	1
164	T1	1	2212	H3	2
180	H3	1	2411	X3	1
190	Beijing	1	2506	LAM1	1
211	LAM3	1	2735	H3	1
239	T2	1	2918	EAI5	1
241	T1	1	3838	EAI5	1
281	T1	1	Orphan or New	T1	1
390	H3	1	Orphan or New ^a^	Unidentified ^a^	21

^a^ Strain with a unique spoligotype pattern not reported in the SITVIT2 database.

**Table 2:** Identification by similarity of orphan strains using the MIRU-VNTRplus database.

Strain	Family	Distance ^a^	Strain	Family	Distance ^a^
INER-02	X	0.093	INER-77	EAI	0.05
INER-17	Haarlem	0.04	INER-79	EAI	0.04
INER-20	S	0.16	INER-80	EAI	0.1
INER-38	LAM	0.093	INER-81	Haarlem	0.09
INER-47	LAM	0.093	INER-84	LAM	0.05
INER-61	Unknown	-	INER-87	Haarlem	0.1395
INER-62	LAM	0.04	INER-88	Uganda I	0.04
INER-64	EAI	0.09	INER-89	Ghana	0.06
INER-70	S	0.093	INER-90	X	0.0698
INER-75	EAI	0.04	INER-92	Haarlem	0.1
			INER-93	LAM	0.14

^a^ A categorical method was used based on the spoligotype patterns for identification by similarity.

**Table 3:** Allelic diversity of the 24 MIRU-VNTR loci among the MTb strains.

Locus	HGDI	Locus	HGDI
MIRU 02	0.5823	Mtub 30	0.6138
Mtub 04	0.4095	ETRB	0.6475
ETRC	0.7244	MIRU 23	0.5657
MIRU 04	0.7167	MIRU 24	0.8221
MIRU 40	0.4488	MIRU 26	0.8689
MIRU 10	0.6114	MIRU 27	0.6211
MIRU 16	0.4345	Mtub 34	0.7691
Mtub 21	0.6655	MIRU 31	0.6957
MIRU 20	0.6197	Mtub 39	0.6639
QUB2163b	0.6627	QUB26	0.7085
ETRA	0.6526	QUB4156	0.5395
Mtub 29	0.7557	MIRU 39	0.5694

## Data Availability

All data derived from this study are provided in the article.
